# MicroRNA-27a contributes to the malignant behavior of gastric cancer cells by directly targeting PH domain and leucine-rich repeat protein phosphatase 2

**DOI:** 10.1186/s13046-017-0516-2

**Published:** 2017-03-21

**Authors:** Lei Ding, Shanyong Zhang, Mu Xu, Renwen Zhang, Pengcheng Sui, Qing Yang

**Affiliations:** 1 Department of Pathogenobiology, College of Basic Medical Sciences, Jilin University, 126 Xinmin Street, Changchun, 130021 Jilin Province China; 2 Department of Orthopedics, The Second Hospital, Jilin University, Changchun, Jilin China

**Keywords:** miR-27a, PHLPP2, Gastric cancer, Proliferation, Metastasis

## Abstract

**Background:**

Accumulating evidence indicates that microRNA-27a (miR-27a) is involved in carcinogenesis and tumor progression. However, the exact function and molecular mechanism of miR-27a in gastric cancer remain unclear.

**Methods:**

Quantitative real-time PCR (qRT-PCR) was used to quantify the expression of miR-27a and its target gene. The function of miR-27a in gastric cancer was investigated through in vitro and in vivo assays (MTT assay, colony formation assay, flow cytometry assay, wound healing assay, migration and invasion assay, immunohistochemistry (IHC), immunofluorescence (IF) and Western blot). A luciferase reporter assay was conducted to confirm the target gene of miR-27a.

**Results:**

We found that miR-27a was commonly overexpressed in gastric cancer and high expression of miR-27a was associated with distant metastasis, lymph node metastasis, advanced T stage and advanced clinical stage. Functional assays demonstrated that overexpression of miR-27a in AGS cells accelerated cell proliferation, migration and invasion and suppressed apoptosis. Meanwhile, opposite results were observed in SGC-7901 cells when miR-27a was suppressed. Consistently, down-regulation of miR-27a inhibited the growth and metastasis of engrafted tumors in vivo. Furthermore, we found PH domain and leucine-rich repeat protein phosphatase 2 (PHLPP2) to be a new target of miR-27a, and downregulation of PHLPP2 could rescue the effect of anti-miR-27a in gastric cancer cells. In addition, miR-27a-mediated suppression of PHLPP2 led to stimulation of the AKT/GSK3β pathway.

**Conclusions:**

Our data suggest that miR-27a functions as a crucial oncogenic miRNA in gastric cancer. It can promote proliferation and metastasis of tumor cells by suppressing PHLPP2 and activating the AKT/GSK3β pathway. Therefore, miR-27a is a potential novel therapeutic target in gastric cancer treatment.

**Electronic supplementary material:**

The online version of this article (doi:10.1186/s13046-017-0516-2) contains supplementary material, which is available to authorized users.

## Background

Gastric cancer (GC) is a common tumor of the digestive system. In 2012, an estimated 951,600 new stomach cancer cases and 723,100 deaths occurred worldwide, and in less developed countries, liver and stomach cancers among men are the second and third most frequently diagnosed cancers, respectively [1]. Although recent advancements in early detection, therapy, and prevention have partially enhanced the survival rate of early-stage GC, stage IV GC is still incurable and has a very poor 5-year survival rate of approximately 4–5% [2]. The unlimited proliferation and strong metastasis capacities of GC tumor cells are the main causes of the high degree of malignancy and the poor overall survival. As we know, high recurrence and metastasis rates have always been the primary obstacle to improve the survival rate of GC. Over 70% of patients experience recurrent and metastatic disease following interventions [3]. Therefore, it is of great significance to further elucidate the molecular mechanisms of GC and improve the current preventive and therapeutic strategies against this disease.

MicroRNAs (miRNAs) are a class of small non-coding, endogenous, single-stranded RNAs of 18–23 nucleotides in length, which regulate gene expression by promoting mRNA degradation or inhibiting translation by binding to the 3′-untranslated region(3′-UTR) of their target mRNAs in a sequence-specific manner [4–6]. It has been reported that more than 60% of protein translation is regulated by miRNAs. Convincing evidence has confirmed that miRNAs regulate a majority of cellular processes related to the biological behavior of tumors, including cell proliferation, apoptosis, differentiation and metastasis [7–9]. Dysregulation of miRNA expression has been found in various types of human cancers [10–14]. Compelling evidence has suggested that miRNAs are novel modulators of tumor progression and new targets for tumor therapy in GC [15, 16]. MicroRNA-27a (miR-27a) is located on chromosome 19 (19p13.1). The role of miR-27a in tumorigenesis differs in various cells and tissues. It is regarded as an oncogene in several types of tumors, such as osteosarcoma [17], laryngeal carcinoma [18] and breast cancer [19]. However, miR-27a is suggested to be a cancer suppressive miRNA in esophageal squamous cell carcinoma [20] and colorectal cancer [21]. Its functions and molecular mechanisms in GC need to be further investigated.

In the current research, we explored the hidden function of miR-27a in the carcinogenesis and development of GC. We identified that miR-27a was upregulated in GC cells and tissues and the increased expression of miR-27a led to the promotion of tumorigenicity and metastasis of GC. As we know, a miRNA exerts its function through its target genes. Here, we explored whether PH domain and leucine-rich repeat protein phosphatase 2 (PHLPP2) was a novel target of miR-27a. PHLPP2, an isoform of the PHLPP, has been reported to induce cell cycle arrest and apoptosis and to suppress tumor metastasis of various types of cancer [22–24]. PHLPP2can directly dephosphorylate and inactivate Akt at Ser473 and subsequently inhibit the PI3K/Akt signaling pathway [25, 26]. Our study suggested that miR-27a exerts its functions of promoting proliferation and metastasis in GC cells by activating the Akt signaling pathway via targeting PHLPP2.

## Methods

### Patients and tissue specimens

In this study, 50 human GC tissues and matched adjacent non-tumor tissues (3 cm from the margin of the resected neoplastic tissues) were obtained from patients who underwent surgical stomach resection between November 2014 and December 2016 in the Second Hospital of Jilin University (Changchun, China). This study was approved by the Ethics Committee of the School of Basic Medical Sciences, Jilin University and prior informed consent was obtained from all patients. The samples were snap-frozen in liquid nitrogen and stored at −80 °C for later RNA extraction or formalin fixed and paraffin-embedded for immunohistochemistry (IHC). The miRNA sequencing data and the corresponding clinical information of the patients with gastric cancer were downloaded from the TCGA data portal. We screened the data and selected those tumor samples with matched non-tumor samples and detailed clinical information for the present study. The clinical features of the patients, including age, gender, tumor size, differentiation status, lymph node metastasis status, distant metastasis status and clinical stage were collected from their medical records and summarized in Table 1.

**Table 1:** Clinical features of all patients included in this study

Clinicopathological features	TCGA database (*n* = 40)	Collected tissue samples (*n* = 50)
Age (year)		
< 60	7	16
≥ 60	33	34
Gender		
Female	16	17
Male	24	33
T Stage		
T1-2	19	13
T3-4	21	37
Nodal stage		
N0	16	13
N1 + N2	24	37
Metastasis		
M0	36	43
M1	2	7
MX	2	0
Grade		
G2	15	19
G3	24	31
GX	1	0
Stage		
I + II	28	20
III + IV	12	30

*MX* metastasis status unknown, *GX* differentiation status unknown

### Cell lines and culture

In this study, the human GC cell lines MGC-803, HGC-27, BGC-823, AGS and SGC-7901 and the normal gastric epithelial cell line GES-1 were obtained from the Cell Bank of Shanghai Institute of Biochemistry and Cell Biology (Shanghai, China). The cells were stored in liquid nitrogen and cultured in RPMI-1640 medium (Gibco, USA) supplemented with penicillin (100 IU/mL), streptomycin (100 mg/mL) and 10% FBS (fetal bovine serum) and maintained at 37 °C in a humidified incubator containing 5% CO_2_.

### RNA isolation and quantitative real-time PCR (qRT-PCR)

Total RNA was isolated from tissue and cell specimens using Trizol (TaKaRa, China), and total RNA was extracted according to the manufacturer’s instructions. The RNA concentration was measured with a BioSpectrometer (Eppendorf, Germany). The RNA samples were reversely transcribed into cDNA using the TransScript RT reagent Kit (TransGen, China). QRT-PCR was performed with FastStart Universal SYBR Green Master (ROX) (Roche, USA). β-actin and U6 were used to normalize the level of mRNA and miRNA expression, respectively. β-actin primers were 5′-CTGGAACGGTGAAGGTGACA-3′ and 5′-AAGGGACTTCCTGTAACAATGCA-3′; PHLPP2 primers were 5′-CCAATGAGCAAGGACAGGAT-3′ and 5′-GGTCCTCTGGTTCCATCTGA-3′. The Bulge-Loop miRNA qRT-PCR Primer kit (RIBOBIO, China) was used for detecting miR-27a expression. QRT-PCR was performed using the CFX96 Real-Time system (Bio-Rad, USA), and the data were analyzed using the 2^∆∆CT^ method.

### Protein extraction and Western blot

Total cellular proteins were extracted using the cell lysis buffer for Western blot. The protein samples were subjected to 10% sodium dodecyl sulfate-polyacrylamide gel electrophoresis (SDS-PAGE) and transferred onto PVDF membranes (Bio-Rad, USA). The membranes were blocked in 5% skim milk and then incubated with a specific primary antibody and a secondary antibody, and they were then detected by enhanced chemiluminescence (ECL). The immunoblots were visualized using the Image Quant LAS 4000 digital imaging system (GE, USA). The following primary antibodies were used: Antibodies for PHLPP2 (PA5-25995) and Vimentin (PA5-2723) were obtained from Thermo Fisher. Antibodies for GSK-3β (ab131356), p-GSK-3β (ab75814), P27 (ab62364), P21 (ab109520), CyclinD1 (ab134175), E-cadherin (ab152102) and Snail (ab82846) were purchased from Abcam. Antibodies for AKT (D260001) and p-AKT (D155022) were purchased from Sangon Biotech . While the β-actin antibody and the secondary antibodies were purchased from Beyotime.

### Immunohistochemistry and immunofluorescence (IF) analysis

Paraffin blocks from GC and normal tissues were sectioned into 4-μM-thick sections. The samples were deparaffinized in xylene and rehydrated using a series of graded alcohol. The slides were blocked with 10% goat serum before incubation with the primary antibody. The samples were incubated overnight with a primary antibody and then with a secondary antibody. For immunofluorescence, cells were seeded in 96-well culture plates, incubated with primary antibodies and then incubated with fluorophore-conjugated secondary antibody. They were visualized using a microscope or an inverted fluorescence microscope TE-2000S (Nikon). 4′6-Diamidino-2-phenylindole (DAPI) and fluorophore-conjugated secondary antibodies were obtained from Beyotime (Shanghai, China).

### Transient transfection

MiR-27a agomirs (miR-27a) and antagomirs (anti-miR-27a) were used for gain-of-function and loss-of-function analyses. MiR-27a agomirs, miR-27a agomirs negative control (NC), miR-27a antagomirs, miR-27a antagomirs negative control, siRNA against PHLPP2 (siPHLPP2) and siRNA negative control were synthesized by Ribobio (Guangzhou, China). Oligonucleotides were transfected into GC cell lines using Lipofectamine RNAiMAX (Invitrogen) according to the manufacturer’s instructions.

### Luciferase reporter assay

The wild-type 3′-UTR fragment of PHLPP2 was amplified by PCR and cloned into the *Xba*I and *EcoR*I sites of the dual-luciferase miRNA target expression vector (Promega, USA), the resulting vector was named wtPHLPP2-3′-UTR. The mutant variant of the PHLPP2 3′-UTR vector was generated from wtPHLPP2-3′-UTR by mutating nucleotides that potentially bind to miR-27a and named mtPHLPP2-3′-UTR. The vectors (wtPHLPP2-3′-UTR or mtPHLPP2-3′-UTR together with miR-27a agomirs or miR27a agomirs NC/miR-27a antagomirs or miR-27a antagomirs NC) were transfected into AGS and SGC-7901 cells using Lipofectamine 2000 reagent (Invitrogen). Luciferase activity was measured 48 h later with a dual-luciferase assay system (Promega) with a Synergy H1 Multi-Mode Microplate Reader (BioTek, USA). Luciferase activity ratios were presented as firefly luciferase values/renilla luciferase values.

### Cell proliferation, colony formation, apoptosis and cell cycle assays

Tumor cell proliferation was assessed using the MTT and colony formation assays according to the manufacturer’s protocol. For the MTT assay, 0.5 × 10^4^ cells per well were seeded in 96-well plates, and the OD490 was measured on days 1, 2, 3, 4 and 5. Five hundred gastric cancer cells per well were seeded in 6-well plates. After two weeks, colonies were fixed with methanol containing 0.2% crystal violet, and the number of colonies was counted. Furthermore, an apoptosis assay was performed 48 h after the transfection of miR-27a antagomirs, miR-27a agomirs or siPHLPP2 into SGC-7901 and AGS cells using the AnnexinV FITC/PI Apoptosis Detection Kit (Roche) and the Accuri C6 flow cytometer (BD, USA). In addition, cell cycle analyses were performed 48 h after the transfection of oligonucleotides into GC cells using a cell cycle detection kit (Keygen, Nanjing, China) and the Accuri C6 flow cytometer (BD, USA).

### In vitro wound healing, tumor cell migration and invasion assays

For the wound healing assay, appropriate GC cells were seeded into 12-well plates, transfected with oligonucleotides and cultured for 1 day. After the cells achieved nearly 90% confluence, a line was scraped with a 10-μl pipette tip, cells were washed with medium until no floating cells were present. Then, the medium in the plates was replaced and the cells were cultured for 24 h. The speed of wound closure was imaged, and the rate of closure was calculated via counting changes of cell covered area for five randomly chosen fields. Tumor cell migration and invasion capacity were assessed using Transwell chambers (Corning, USA) in 24-well plates. In brief, tumor cells were transfected with the oligonucleotides, and 24 h later, they were resuspended in serum-free RPMI 1640 medium and seeded into the upper chamber of Transwells whose membrane was coated or not with or matrigel (BD, USA), while the lower chamber was filled with fresh medium containing 10% FBS. After 24 h of culture at 37 °C, the cells remaining in the top chambers were taken away carefully using cotton swabs, while the cells migrating and invading the lower surface of the chambers were fixed and stained with a solution containing 0.1% crystal violet (Beyotime, Shanghai, China) and 20% methanol, reviewed and photographed under a microscope at × 200 magnification in five random fields. The quantity of migrating and invading cells was assessed by counting together five different fields of view under an optical microscope.

### Tumor xenograft model

Six- weeks old BALB/c nude mice (*n* = 12) were purchased from the Laboratory Animal Research Institute of the Beijing Chinese Academy of Medical Sciences and housed under specific pathogen-free conditions. All animal experiments were undertaken in accordance with the National Institute of Health Guide for the Care and Use of Laboratory Animals, with the approval of the Scientific Investigation Board of the College of Basic Medicine, Jilin University. The nude mice received the subcutaneous injection of SGC-7901 cells into the flank. When the tumor volume reached 100 mm^3^, miR-27a antagomir or NC were inoculated into the xenograft tumor by multi-point injection once every 2 days, and these mice were closely observed for tumor growth. Tumor size was measured every 2 days with a digital caliper. The tumor volume was calculated using the formula: length × width^2^ × 0.5. Then, all mice were sacrificed, and tumors were removed, photographed and weighed. Tumor grafts from the nude mice were fixed with a formaldehyde solution and embedded in paraffin for IHC analysis. For the metastasis assay, the cells (2 × 10^6^ cells per mouse) were vaccinated to the spleens of the mice. Two weeks later, miR-27a antagomir or NC were administered through the tail vein of each group respectively. Six weeks later nude mice were sacrificed. Then the livers of mice were removed and fixed with formaldehyde solution and embedded with paraffin. The paraffin fixed tissues were serially sectioned and stained by hematoxylin-eosin (HE) staining to identify metastatic nodules.

### Statistical analysis

All statistical analyses were performed using SPSS 18.0 (SPSS Inc., Chicago, USA) and GraphPad Prism 6 (GraphPad Software Inc., CA, USA) software. Differences in the level of expression of miR-27a and PHLPP2 between GC and the corresponding normal tissues were evaluated using the paired *t*-test. The association of expression of miR-27a and PHLPP2 with clinical parameters was analyzed using the unpaired *t*-test. Correlation of miR-27a expression with that of PHLPP2 was conducted with the Pearson correlation test. For in vitro experiments, the *t*-test was used to analyze the difference between two groups. All *P*-values were two sided, and *P* < 0.05 was considered to be statistically significant. All data are presented as the mean ± standard deviation (SD) from at least three independent replicates.

## Results

### MiR-27a is upregulated in gastric cancer tissues and cell lines

**Fig. 1:**
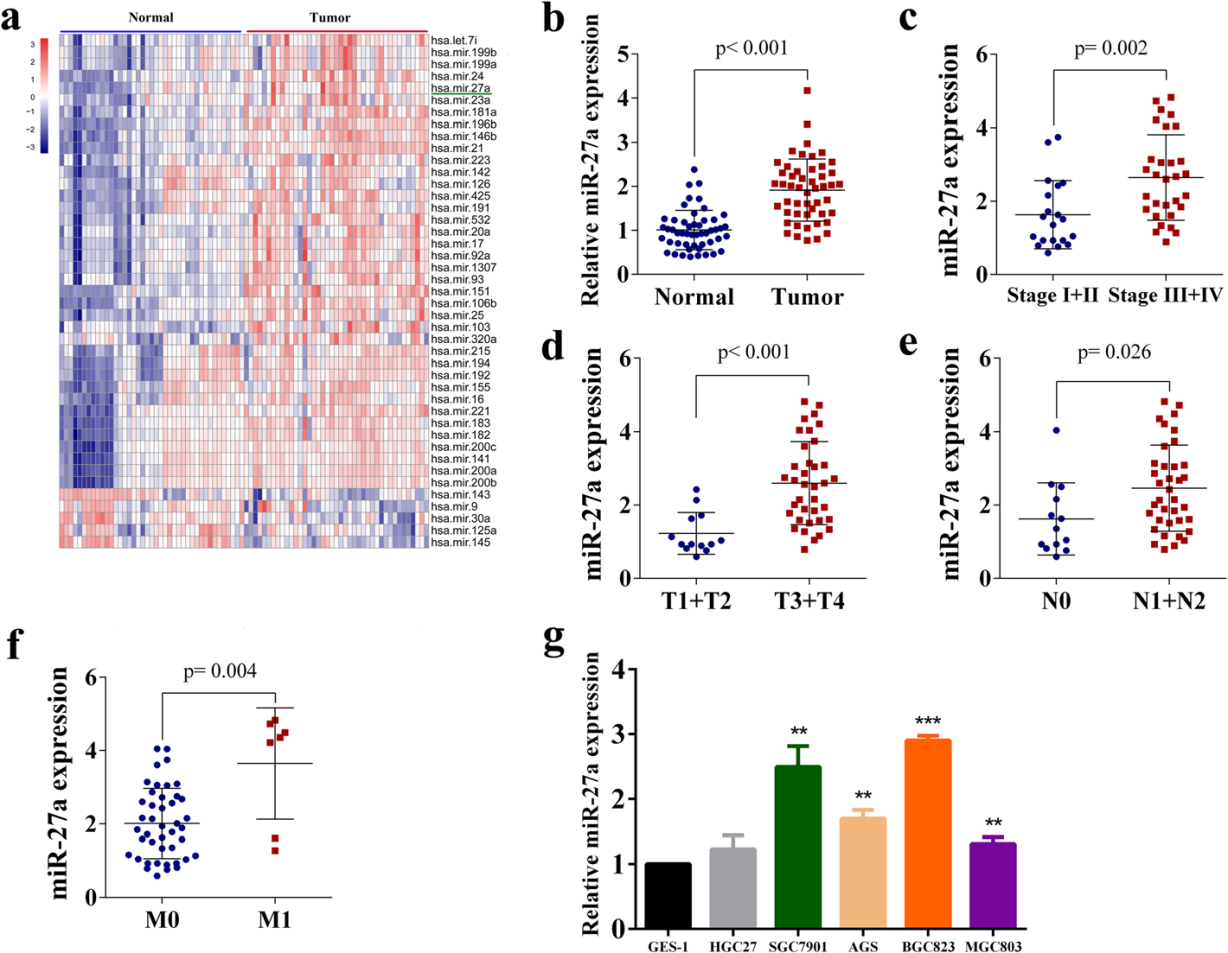
Expression of miR-27a in gastric cancer tissues and cells. **a** Hierarchical cluster heat map of differentially expressed miRNAs in gastric cancer and corresponding normal tissues generated from miRNA sequencing data from the TCGA database. *Red* in the heat map denotes upregulation; *blue* denotes downregulation. The *green* arrow indicates miR-27a. **b** Upregulation of miR-27a in gastric cancer. *P* < 0.001.qRT-PCR analysis of miR-27a expression in 50 pairs of gastric cancer and corresponding normal tissues. MiR-27a expression was normalized to U6. **c** MiR-27a expression in gastric cancers at different clinical stages. *P* = 0.002. **d** MiR-27a expression in gastric cancers at different T stages. *P* < 0.001. **e** MiR-27a expression in gastric cancers at different N stages. *P* = 0.026. **f** MiR-27a expression in gastric cancers at different M stages. *P* = 0.004. **g** MiR-27a expression in gastric cancer cell lines (HGC, SGC-7901, AGS, BGC and MGC-803) compared with normal gastric epithelial cells GES-1 detected using qRT-PCR. **P < 0.01 and ***P < 0.001

Sequencing data downloaded from the TCGA database showed altered miRNAs expression in gastric cancer tissues compared to the corresponding normal tissues, miR-27a was marked with green underline (Fig. 1a). We detected miR-27a expression in 50 tumor tissues and their paired non-tumor tissues by qRT-PCR. The mean expression level of miR-27a was higher in tumor tissues compared with matched non-tumor tissues (Fig. 1b). We also found that a higher miR-27a level was associated with advanced clinical stage (Fig. 1c), advanced T stage (Fig. 1d), advanced N stage (Fig. 1e) and advanced M stage (Fig. 1f). Furthermore, we compared a normal gastric cell line (GES-1) with a panel of GC cell lines (MGC-803, HGC-27, BGC-823, AGS, and SGC-7901) for the expression of miR-27a and found generally increased miR-27a expression in gastric cancer cell lines (Fig. 1g). These results indicated that ectopic expression of miR-27a is a common event in GC tissues and GC cells. Because the expression level of miR-27a in SGC-7901 and AGS was relatively higher or lower and the transfection efficiency of these two cell lines was higher comparing with other GC cell lines, we chose SGC-7901 and AGS cell lines as models for this study.

### Biological effects of miR-27a on the regulation of cell viability, apoptosis and cell cycle distribution of gastric cancer cells

**Fig. 2:**
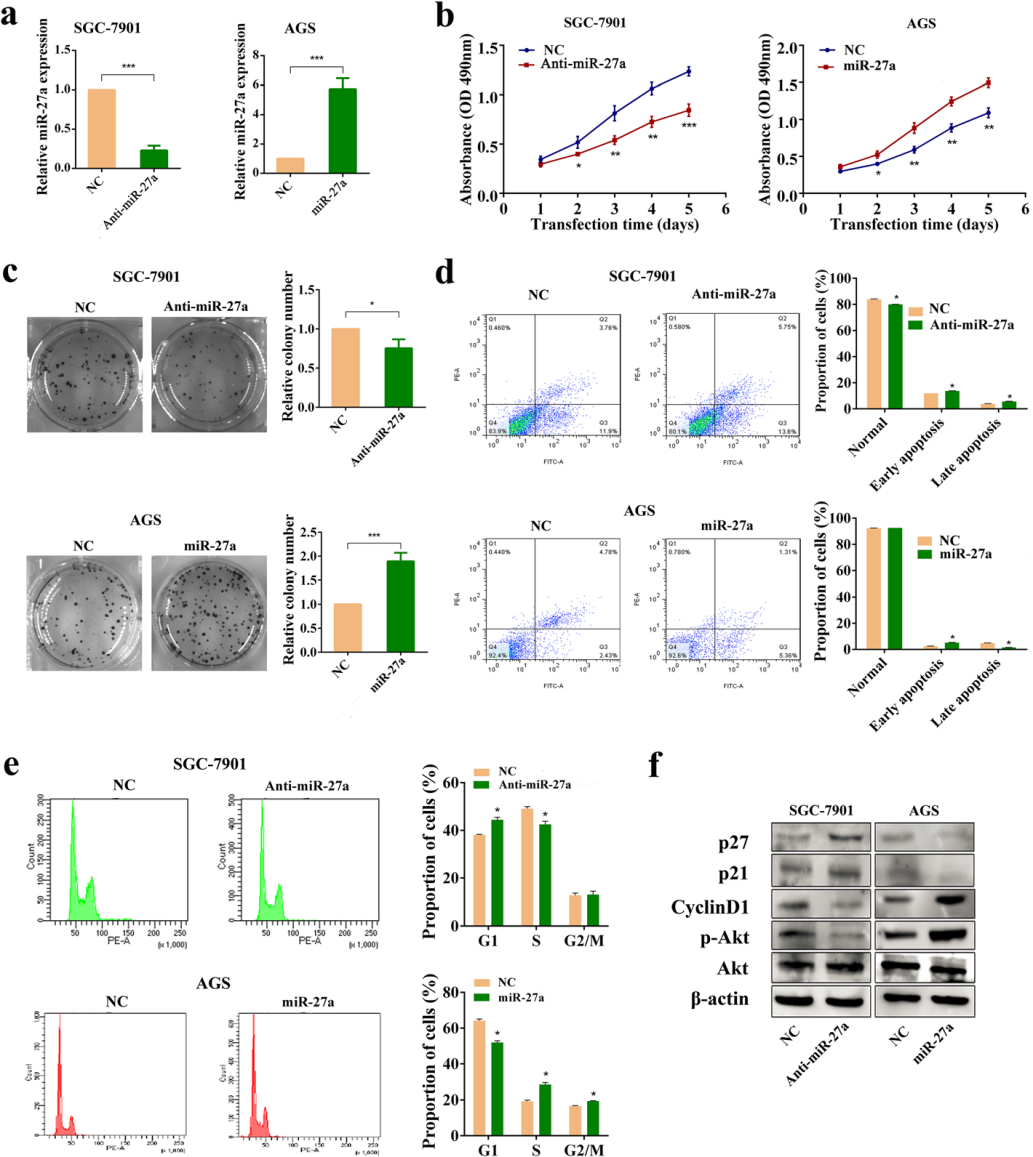
MiR-27a affects viability, cell cycle and apoptosis of gastric cancer cells. **a** QRT-PCR analysis of miR-27a expression in SGC-7901/AGS cells transfected with miR-27a antagomir /miR-27a agomir. **b** Cell viability assay. SGC-7901 cells transfected with miR-27a antagomir and AGS cells transfected with miR-27a agomir were subjected to MTT assay 24 h after transfection. *P < 0.05, **P < 0.01 and ***P < 0.001. **c** Effects of miR-27a on colony formation of GC cells. SGC-7901 cells transfected with antagomir and AGS cells transfected with miR-27a agomir were seeded onto 6-well plates. The number of colonies was counted on the 14^th^ day after seeding. Representative micrographs (left) and relative quantification analysis of colonies (right). *P < 0.05 and ***P < 0.001. **d** Flow cytometric cell apoptosis assay. Histograms depict the proportions of normal, early apoptosis and late apoptosis in SGC-7901/AGS cells transfected with miR-27a antagomir/agomir. *P < 0.05. **e** Flow cytometric cell cycle distribution assay. Histograms depict the proportions of SGC-7901/AGS cells in G1, S, and G2/M phases after transfection with miR-27a antagomir/agomir. *P < 0.05. **f** Western blot analysis of Akt, p-Akt, p21, p27 and CyclinD1 expression in SGC-7901 and AGS cells in which miR-27a was upregulated and downregulated, respectively

To further explore the function of miR-27a in GC, SGC-7901 cells, which expressed a high level of miR-27a, were transfected with miR-27a antagomirs or their NC. In addition, AGS cells, which presented a relatively low level of miR-27a, were transfected with miR-27a agomirs or their NC. As indicated in Fig. 2a, the transfection successfully affected the expression of miR-27a. SGC-7901 cells transfected with antagomirs displayed cell growth inhibition, while AGS cells transfected with agomirs displayed cell growth induction (Fig. 2b). Colony formation assays indicated that the number of colonies in SGC-7901 cells transfected with antagomirs group was lower than the control (Fig. 2c up panel). Contrary results were founded in AGS cells transfected with agomirs (Fig. 2c down panel). We also carried out apoptosis analysis, and found that down-regulation of miR-27a induced apoptosis in SGC-7901 cells, while overexpression of miR-27a inhibited apoptosis in AGS cells (Fig. 2d). Moreover, we studied whether miR-27a has any effect on the GC cell cycle. MiR-27a down-regulation arrested the tumor cells in the G1 phase and decreased the proportion of cells in S phase, while upregulation of miR-27a had opposite results (Fig. 2e). We further performed Western blot and evaluated the effect of miR-27a on proteins related to cell cycle. The result showed that upregulation of miR-27a in AGS cells increased the level of CyclinD1 and decreased the level of p21 and p27, whereas knockdown of miR-27a in SGC-7901 cells decreased expression of CyclinD1 and increased p21 and p27 levels (Fig. 2f). Altogether, our results indicate that miR-27a is relevant to cell viability and modulates cell cycle regulators such as p21, p27 and CyclinD1.

### MiR-27a regulates migration and invasion in gastric cancer cells

**Fig. 3:**
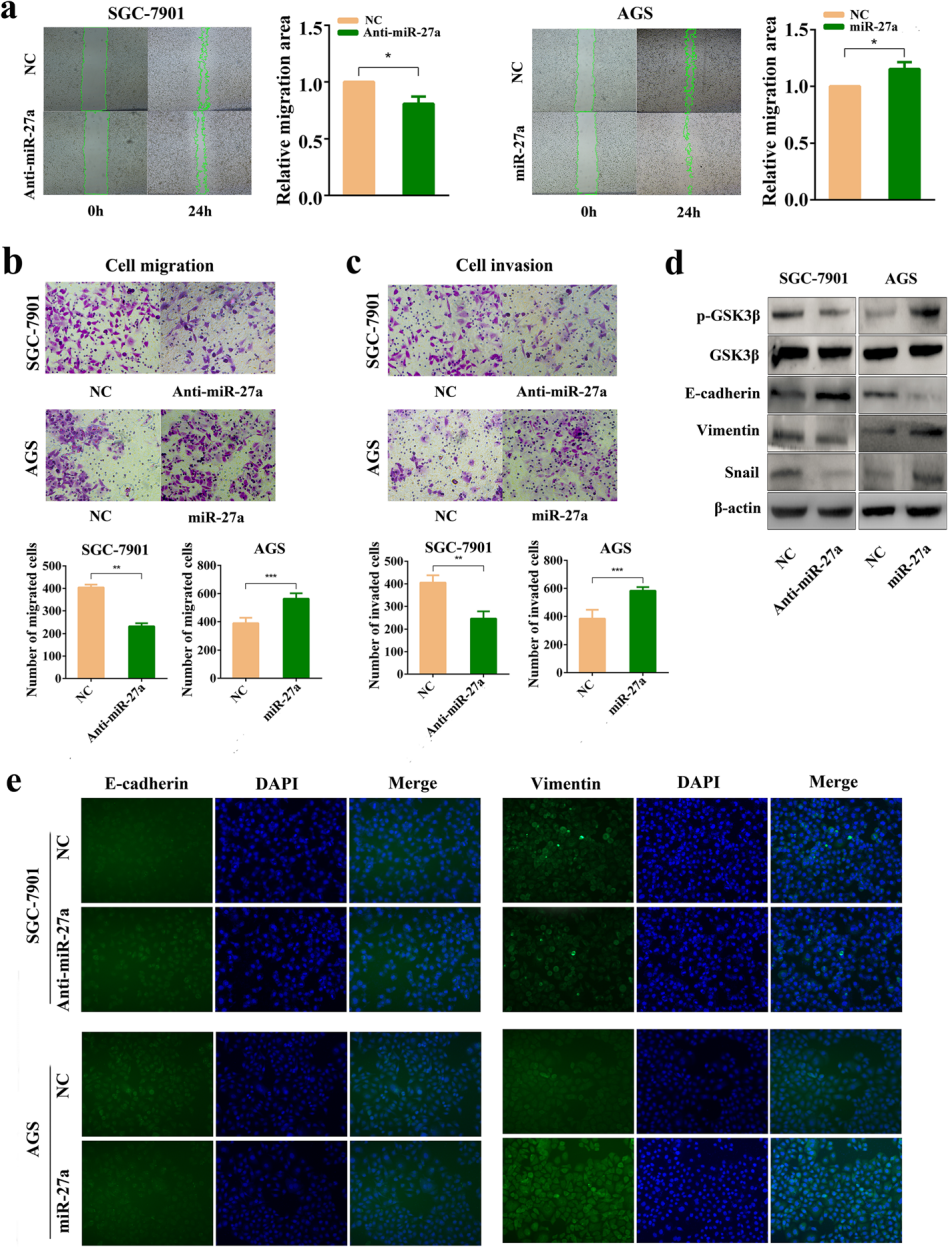
MiR-27a affects migration and invasion of gastric cancer cells. **a** Analysis of migration by wound healing assays. Representative images at 0 and 24 h (right panel). Quantification of the migration area of cells in wound healing assays (left panel). *P < 0.05. **b** Tumor cell Transwell migration assay. Representative images (up panel) and quantification (down panel) of SGC-7901 and AGS cells migration after 24 h transfection with miR-27a antagomir or miR-27a agomir. **P < 0.01 and ***P < 0.001. **c** Tumor cell Transwell invasion assay. Representative images (upper panel) and quantification (lower panel) of SGC-7901 and AGS cells invasion after 24 h transfection with miR-27a antagomir or miR-27a agomir. **P < 0.01 and ***P < 0.001. **d** Western blot analysis of GSK3β, p-GSK3β, Snail, E-cadherin and Vimentin expression in SGC-7901 and AGS cells where miR-27a was downregulated and upregulated, respectively. **e** Immunofluorescence staining analysis of E-cadherin and Vimentin in SGC-7901 and AGS cells in which miR-27a was downregulated and upregulated, respectively

We then explored the potential function of miR-27a in regulating the metastasis capability of GC cells. Wound healing assays showed that lower expression of miR-27a related to slower rate of wound healing and higher expression of miR-27a related to faster healing (Fig. 3a). Transwell assays showed that miR-27a down-regulation impaired the migration/invasion capacity of SGC-7901 cells, and miR-27a upregulation promoted the migration/invasion capacity of AGS cells (Fig. 3b, c). Epithelial–mesenchymal transition (EMT) is an important step during tumor metastasis. We therefore explored the role of miR-27a in EMT. Western blot assays indicated that the expression of Snail and Vimentin decreased in SGC-7901 cells transfected with miR-27a antagomir, while that of E-cadherin increased. Opposite effects were found when AGS cells were transfected with miR-27a agomir (Fig. 3d). Immunofluorescence staining confirmed the ability of miR-27a to suppress E-cadherin expression and to increase the abundance of Vimentin (Fig. 3e). In addition, overexpression of miR-27a induced EMT-related morphological changes in AGS cells (Additional file 1: Figure S1). Together, these data suggests that miR-27a regulates the metastasis process in GC.

### MiR-27a directly targets PHLPP2 by binding to its 3′-UTR to inhibit PHLPP2 expression

**Fig. 4:**
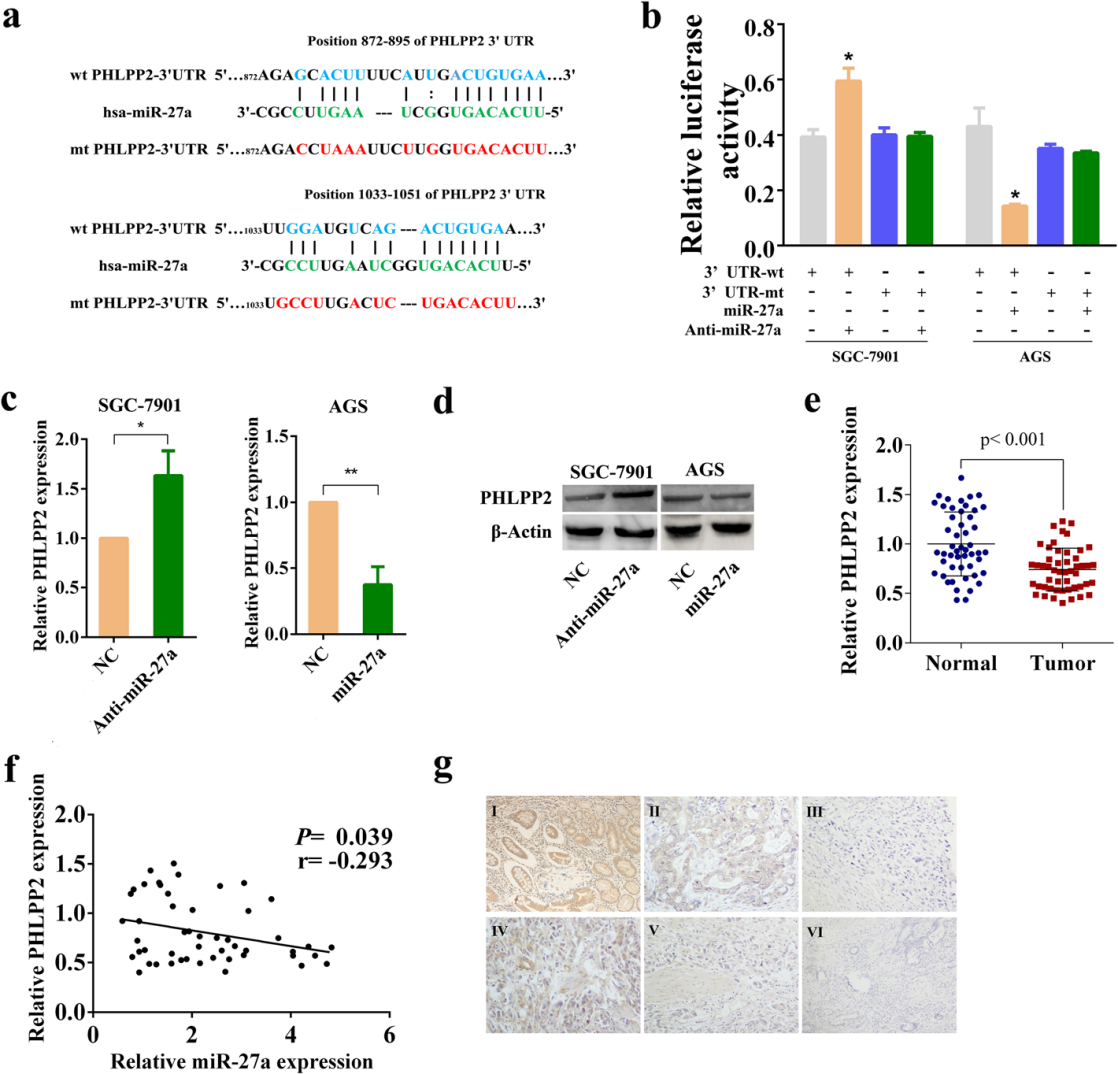
MiR-27a suppresses PHLPP2 expression by directly targeting the PHLPP2 3′-UTR. **a** Sequence alignment of miR-27a and its predicted binding sites (green) in PHLPP2 3′-UTR. Predicted miR-27a target sequence (blue) in the 3′-UTR of PHLPP2 (wtPHLPP2-3′-UTR) and position of mutated nucleotides (red) in the 3′-UTR of PHLPP2 (mutPHLPP2-3′-UTR). **b** Luciferase reporter assay. A vector containing wild type PHLPP2 3′-UTR or mutant PHLPP2 3′-UTR was co-transfected into GC cells together with indicated oligonucleotides. Luciferase activity ratio was presented as firefly luciferase value/renilla luciferase value. **c** PHLPP2 mRNA levels were detected by qRT-PCR in SGC-7901 and AGS cells transfected with miR-27a antagomir or miR-27a agomir, respectively. **d** Western blot analysis of PHLPP2 expression. PHLPP2 protein levels were detected by Western blot in SGC-7901 and AGS cells transfected with miR-27a antagomir or miR-27a agomir, respectively. **e** Relative expression of PHLPP2 in gastric cancer tissues. PHLPP2 expression was tested through qRT-PCR in 50 paired samples. Expression of PHLPP2 mRNA in each tumor tissue was normalized to its matched normal tissue. **f** Pearson correlation analysis between miR-27a and PHLPP2 mRNA levels in human gastric cancer tissues. (r = -0.293, *P* = 0.039). **g** Immunohistochemistry. PHLPP2 protein expression in gastric cancer tissues was analyzed by immunohistochemistry. I, adjacent normal tissue; II, moderately differentiated adenocarcinoma; III, poor differentiated adenocarcinoma; IV, mucous layer infiltration; V, muscular layer infiltration; VI, whole layer infiltration (×200 magnification)

To investigate the hidden molecular mechanism by which miR-27a modulates GC cells, we then investigated target genes of miR-27a. We utilized different public bioinformatics tools (TargetScan, StarBase V2.0 and miRDB) to search for the theoretical target genes and the binding sites [27]. Among the predicted targets, PHLPP2 was chosen for further analysis because of its vital role in inhibiting tumor development and modulating cell proliferation and metastasis [28, 29]. Putative target sites of miR-27a in 3′-UTR of PHLPP2 were shown in Fig. 4a. Dual luciferase assays showed an increment of luciferase activity in SGC-7901 cells transfected with miR-27a antagomir compared to the negative control. In contrast, we obtained the opposite results in AGS cells transfected with miR-27a agomir (Fig. 4b). To determine whether miR-27a down-regulated PHLPP2 at the mRNA or protein level, we examined PHLPP2 expression by qRT-PCR and Western blot. As shown in Fig. 4c, expression of PHLPP2 mRNA was upregulated in SGC-7901 cells transfected with miR-27a antagomir. Conversely, the mRNA level of PHLPP2 was decreased in AGS cells transfected with miR-27a agomir. Western blot analyses showed that the protein levels of PHLPP2 were also upregulated after inhibition of miR-27a in SGC-7901 cells, whereas PHLPP2 was downregulated in AGS cells overexpressing miR-27a (Fig. 4d). Taken together, these data indicate that PHLPP2 is a direct target gene of miR-27a.

### PHLPP2 expression decreases in gastric cancer tissues and is inversely correlated with miR-27a levels

The result of PHLPP2 expression in gastric cancer tissues by qRT-PCR showed that PHLPP2 mRNA levels were reduced in GC tissues compared to adjacent normal tissues (Fig. 4e). lower expression of PHLPP2 was associated with advanced clinical stage (Additional file 1: Figure S2a), advanced T stage (Additional file 1: Figure S2b), advanced N stage (Additional file 1: Figure S2c) and advanced M stage (Additional file 1: Figure S2d). In addition, a remarkable relationship between miR-27a level and PHLPP2 level was observed in these tumor samples (*r* = −0.293, *P* = 0.039) (Fig. 4f). IHC analysis also showed that PHLPP2 expression was lower in GC tissues than in non-tumor tissues and that high malignant-degree tumor tissues have lower PHLPP2 expression (Fig. 4g).

### MiR-27a exerts its function by suppressing PHLPP2 expression

**Fig. 5:**
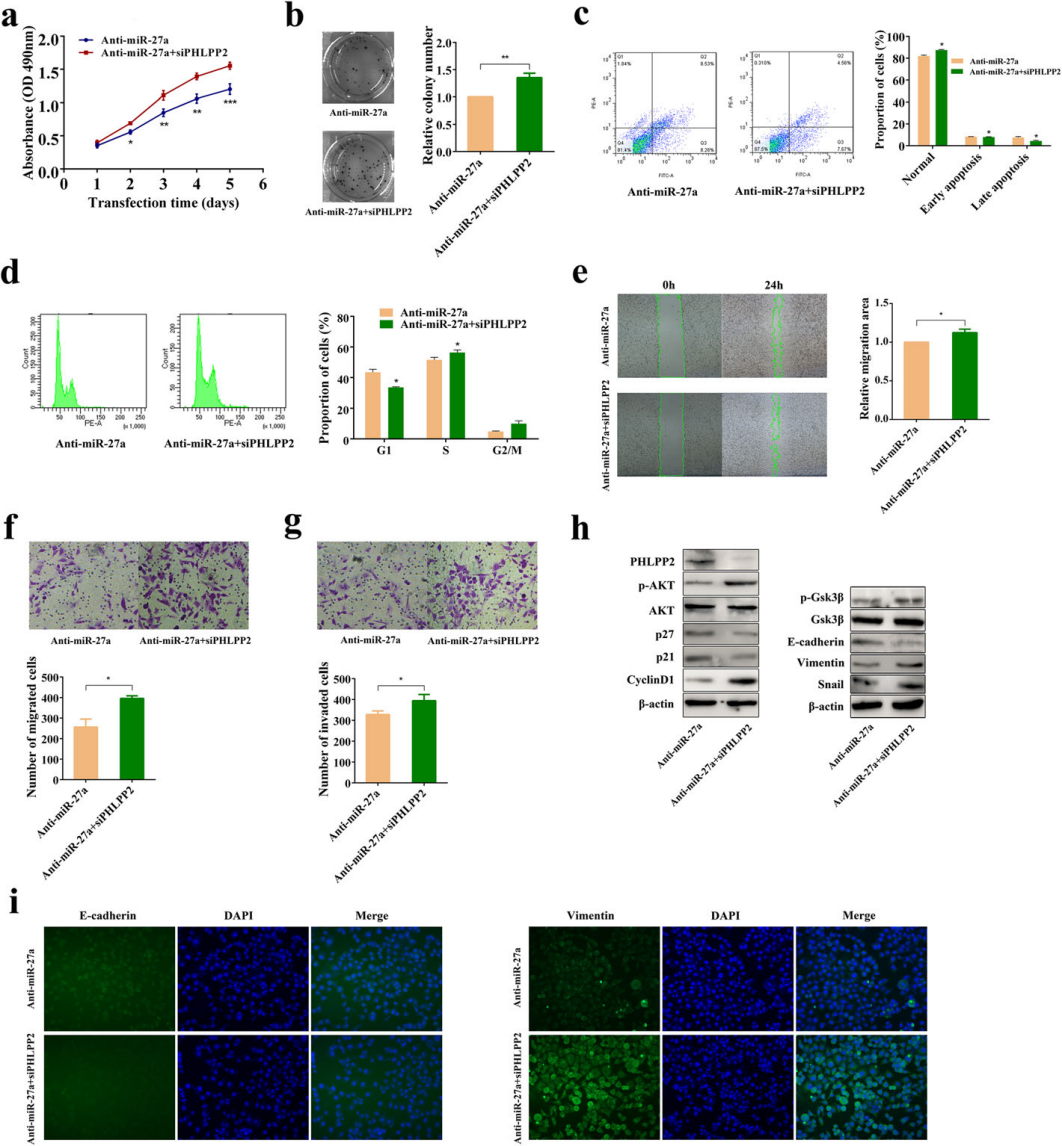
PHLPP2 mediates the effects of miR-27a in GC cells. **a** MTT assay. Cell viability was assessed after PHLPP2 siRNA was transfected into anti-miR-27a SGC-7901 cells. *P < 0.05, **P < 0.01 and ***P < 0.001. **b** Colony formation assay. Transfection of PHLPP2 siRNA in anti-miR-27a SGC-7901 cells promoted cell colonies formation. **P < 0.01. **c** Apoptosis assay. Apoptosis analysis via flow cytometry was performed after transfection of PHLPP2 siRNA into the anti-miR-27a SGC-7901 cells. *P < 0.05. **d** Flow cytometry cell cycle assay. Cell cycle distribution was analyzed after transfection of PHLPP2 siRNA into anti-miR-27a SGC-7901 cells. *P < 0.05. **e** Analysis of the migration with the wound healing assays. *P < 0.05. **f** Tumor cell migration assay. Representative images and quantification of migration level in anti-miR-27a SGC-7901 cells 24 h after transfection with PHLLPP2 siRNA. *P < 0.05. **g** Tumor cell invasion assay. Representative images and quantification of invasion level in anti-miR-27a SGC-7901 cells 24 h after transfection with PHLLPP2 siRNA. *P < 0.05. **h** Western blot. Expression of PHLPP2 and several key proteins in the Akt/GSK3β pathway was detected after transfection of PHLPP2 siRNA into anti-miR-27a SGC-7901 cells. **i** Immunofluorescence staining of E-cadherin (*green*), Vimentin (*green*) and DAPI (*blue*) after transfection of PHLPP2 siRNA into anti-miR-27a SGC-7901 cells

We next examined the role of miR-27a-mediated inhibition of PHLPP2 in the development and maintenance of malignant phenotypes of GC cells. To examine whether miR-27a exerts its function via PHLPP2, SGC-7901 cells were co-transfected with miR-27a antagomir and PHLPP2 siRNA. As indicated in Additional file 1: Figure S3, the transfection with PHLPP2 siRNA successfully decreased the expression of PHLPP2 in co-transfected SGC-7901 cells. MTT and cell colony assays indicated that cell growth inhibition induced by miR-27a knockdown was relieved by transfection with PHLPP2 siRNA (Fig. 5a, b). Flow cytometry analysis showed that the apoptosis and G1-phase arrest caused by miR-27a inhibition were also attenuated by PHLPP2 silencing (Fig. 5c, d). In addition, wound healing and Transwell assays showed that the effect of miR-27a inhibition on cell migration and invasion was abolished by PHLPP2 silencing (Fig. 5e, f, g).

### MiR-27a activates the Akt/GSK3β pathway through PHLPP2 to exert its biologic effects on GC cells

Previous studies showed that PHLPP2 acts as a tumor suppressor gene by modulating a number of critical tumor progression regulators, and it has been reported that PHLPP2 functions through an Akt-independent pathway [25]. Thus, we examined the expression of the components of the AKT pathway by Western blot. Western blot revealed that down-regulation of PHLPP2 abolished the anti-miR-27a mediated regulation of Akt phosphorylation and modulation of the cell cycle regulators p21, p27 and CyclinD1 (Figs. 2f, 5h). In addition, we observed that decreased expression of PHLPP2 could rescue cell migration and invasion impaired by miR-27a knockdown. Therefore, we hypothesized that mi-27a can also promote EMT by targeting PHLPP2 in GC. Western blot showed that silencing PHLPP2 abolished the effect of anti-miR-27a on p-GSK3β, E-cadherin, Vimentin and Snail (Figs. 3d, 5h). Immunofluorescence showed similar results on E-cadherin and Vimentin expression (Fig. 5i). Taken together, these findings confirm that miR-27a promotes GC cell proliferation and metastasis by regulating PHLPP2-mediated activation of the Akt/GSK3β pathway.

### Down-regulation of miR-27a suppresses tumor growth and metastasis of GC in vivo

**Fig. 6:**
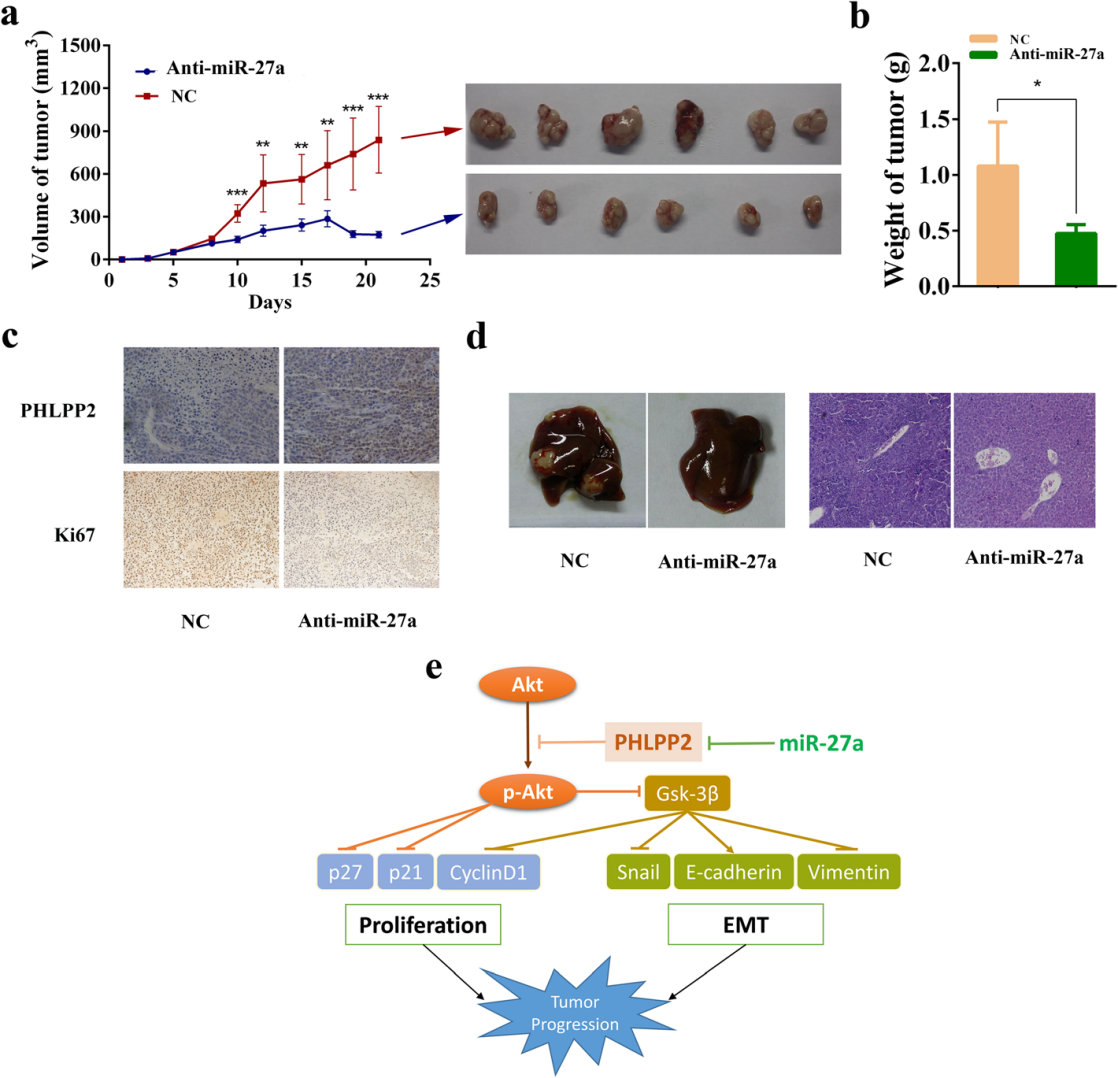
Downregulation of miR-27a inhibits tumor growth and metastasis in vivo. **a** Time-dependent tumor volumes in the negative control and the miR-27a antagomir groups. **P < 0.01 and ***P < 0.001. Photographs of tumors derived from nude mice in the NC and the miR-27a antagomir groups. **b** Average weight of tumors from nude mice. *P < 0.05. **c** Representative photographs of immunohistochemical analysis of Ki-67 and PHLPP2 in tumors from nude mice. **d** Macroscopic observation in the livers and representative images of liver metastases (H&E staining). **e** Schematic presentation of miR-27a and PHLPP2 in the progression of gastric cancer

Considering downregulation of miR-27a suppresses GC cell proliferation and metastasis in vitro, we next generated a xenograft model to identify whether miR-27a was a tumor activator both in vitro and in vivo. As described in the Methods section, SGC-7901 cells were subcutaneously injected into the dorsal flank of nude mice, and then miR-27a antagomir or antagomir control were inoculated into the tumor. Tumors of the anti-miR-27a group grew more slowly than those of the NC group. A decrease in the size of tumors excised from animals of the miR-27a antagomir group was observed compared with that of the control group (Fig. 6a). The average weight of tumors derived from the anti-miR-27a group was also lower than that of tumors derived from the NC group (Fig. 6b). MiR-27a expression of xenografts was detected by qRT-PCR, significant decrease was observed in anti-miR-27a group (Additional file 1: Figure S3). IHC assay confirmed that the tumors of the control group displayed higher Ki-67 expression than the tumors of the miR-27a antagomir group. In addition, the tumors of the control group displayed lower levels of PHLPP2 than the tumors from the miR-27a antagomir group (Fig. 6c). While in the metastasis experiment, macroscopic observation and histological analyses of their livers showed that downregulation of miR-27a inhibited metastasis in the organs (Fig. 6d). Collectively, these data showed that down-regulation of miR-27a resulted in inhibition of GC progression in vivo.

## Discussion

MiRNAs are a class of small RNAs that play an important role in regulating cellular activities in various living systems. They can profoundly affect the expression of a large number of genes that encode proteins. The precise function of individual miRNAs and their mRNA partners in normal and diseased tissues are being extensively investigated. MiRNAs have also been widely reported to act as essential regulators in tumors [4, 5, 16]. MiR-27a is one of the well-identified miRNAs in cancers, and its function is diverse, depending on the cancer types. In some tumors, it plays a role in promoting cancer, while in others it plays a role in tumor suppression [17–21]. Here, we confirmed that miR-27a was highly expressed in GC samples and cells. MiR-27a level was positively associated with the malignant degree of GC. Therefore, we explored the mechanism by which miR-27a influences the carcinogenesis of GC.

Functional in vitro studies suggested that overexpression of miR-27a significantly promoted growth, migration and invasion of GC cells. Furthermore, *our* xenograft mouse model also unveiled the suppressive effects of miR-27a knockdown on tumor growth and metastasis in vivo. Consistent with the data from the biological experiments, we found that the expression level of miR-27a was related to clinic-pathological characteristics, and the upregulated miR-27a expression was associated with distant metastasis, lymph node metastasis and advanced clinical stage. In review of the previous published literature, Zhou L et al. and Zhao X et al. also studied the role of miR-27a in GC. They reported that miR-27a promotes the proliferation of GC cells that is consistent with the result in our present study [30, 31]. In addition, we further studied the prometastatic effect of miR-27a in GC, and reported a new target gene of miR-27a.

In general, miRNAs perform their functions via suppression of specific target genes. To find a novel target through which miR-27a exerts its effects in GC, we employed an integrated approach using public bioinformatics tools. PHLPP2 was predicted as a direct target of miR-27a using bioinformatics algorithms. We next verified this prediction via qRT-PCR, Western blot and dual luciferase report assays. We found that the mRNA expression of PHLPP2 was significantly down-regulated in GC patient specimens and it was inversely associated with miR-27a levels. The levels of PHLPP2 mRNA and protein were reduced upon the upregulation of miR-27a in AGS cells, while the levels of PHLPP2 mRNA and protein were increased upon the down-regulation of miR-27a in SGC-7901 cells. As a member of the Ser/Thr protein phosphatase family, PHLPP2 is critical for suppressing cell survival by negatively modulating the signaling pathways stimulated by AKT, PKC, MAPK and Mst1 [32–36]. To confirm whether PHLPP2 is a target that mediates the function of miR-27a, we performed a loss of function approach to functionally characterize PHLPP2 in growth and metastasis of GC. We found that PHLPP2 silencing can partially attenuate the effects produced by miR-27a inhibition on cell proliferation, apoptosis, migration and invasion. Studies have shown that PHLPP2 can directly dephosphorylate AKT to inhibit its signaling activity [25, 26]. Our results revealed that p-AKT and CyclinD1 expression was down-regulated, p21 and p27 was upregulated in SGC-7901 cells where miR-27a was suppressed, while PHLPP2 silencing could abolish these changes. These findings indicate that the PHLPP2/AKT axis contributes to the miR-27a-mediated progression of GC. Meadow’s study found that AKT could promote EMT by suppressing the GSK3β kinase activity [37]. Phosphorylated and inactivated GSK3β can promote the activity of Snail, further inhibiting E-cadherin expression and resulting in EMT [38, 39]. Cancer cell metastasis is highly related to EMT process. Aberrant expression of E-cadherin, Vimentin and Snail is associated with EMT and tumor metastasis [40, 41]. In this study, we found that the increase in the expression of miR-27a was associated with higher frequency of lymph node metastasis and distant metastasis in the patients with gastric cancer. By examining the expression of E-cadherin, Vimentin, Snail and p-GSK3β, we demonstrated that miR-27a might promote EMT through an Akt/GSK3β dependent pathway. Some previous studies also reported that miR-27a is related to cancer metastasis. Pan W et al. showed that miR-27a contributes to metastatic properties of osteosarcoma cells by targeting MAP2K4 [42]. Li J et al. found that miR-27a promotes lung adenocarcinoma cells metastasis by interacting with RKIP [43]. Here we found that miR-27a promoted gastric cancer cells metastasis by interacting PHLPP2, which is novel finding and has not been reported previously to the best of our knowledge.

## Conclusions

In summary, our current study demonstrated that miR-27a possessed tumor inducing effects on gastric cancer through the novel target PHLPP2. Downregulation of miR-27a suppressed multiple malignant biological behaviors, including inhibition of tumor cell growth, and reduction of tumor cell migration and invasion. In addition, silencing of PHLLP2 could abolish malignant biological behaviors changes induced by inhibition of miR-27a. Conclusively, our findings indicated that miR-27a acted as an oncogene in GC and clarified its functional mechanism. Further studies will confirm if miR-27a could actually be used as a novel target for GC prevention and therapy.

## Additional file

Additional file 1: Figure S1. Morphological changes of AGS cells induced by miR-27a Overexpression. **Figure S2.** Associations between PHLPP2 expression and clinicopathological features. **Figure S3.** Relative PHLPP2 mRNA expression level in SGC-7901 cells co-transfected with miR-27a antagomir and PHLPP2 siRNA. **Figure S4.** Relative expression of miR-27a in subcutaneously trans-planted tumor tissues. (DOCX 8407 kb)
