# Supplementary material for: MicroRNA-27a contributes to the malignant behavior of gastric cancer cells by directly targeting PH domain and leucine-rich repeat protein phosphatase 2
Source: J Exp Clin Cancer Res. 2017 Mar 21;36:45. doi: 10.1186/s13046-017-0516-2 (PMC5361803; doi:10.1186/s13046-017-0516-2)
Supplement: Additional file 1: Figure S1. — Morphological changes of AGS cells induced by miR-27a Overexpression. Figure S2. Associations between PHLPP2 expression and clinicopathological features. Figure S3. Relative PHLPP2 mRNA expression level in SGC-7901 cells co-transfected with miR-27a antagomir and PHLPP2 siRNA. Figure S4. Relative expression of miR-27a in subcutaneously trans-planted tumor tissues. (DOCX 8407 kb) [file 13046_2017_516_MOESM1_ESM.docx]

**Additional file:**

**Figure S1:**

**
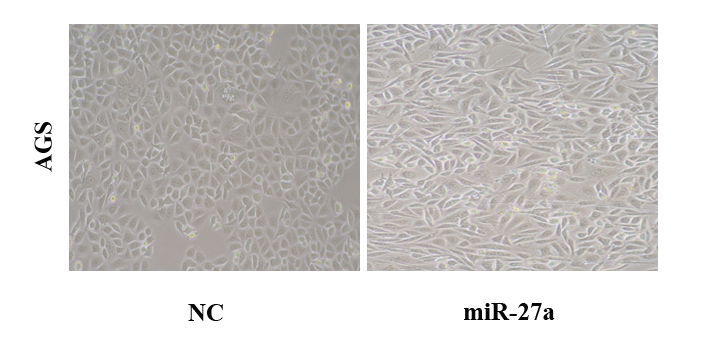
**

**Figure S1.** **Morphological changes of AGS cells induced by miR-27a Overexpression.** AGS cells were transfected with miR-27a agomirs and miR-27a agomirs negative control 48h later, cell morphologies were documented using an inverted microscope.

**Figure S2:**

**
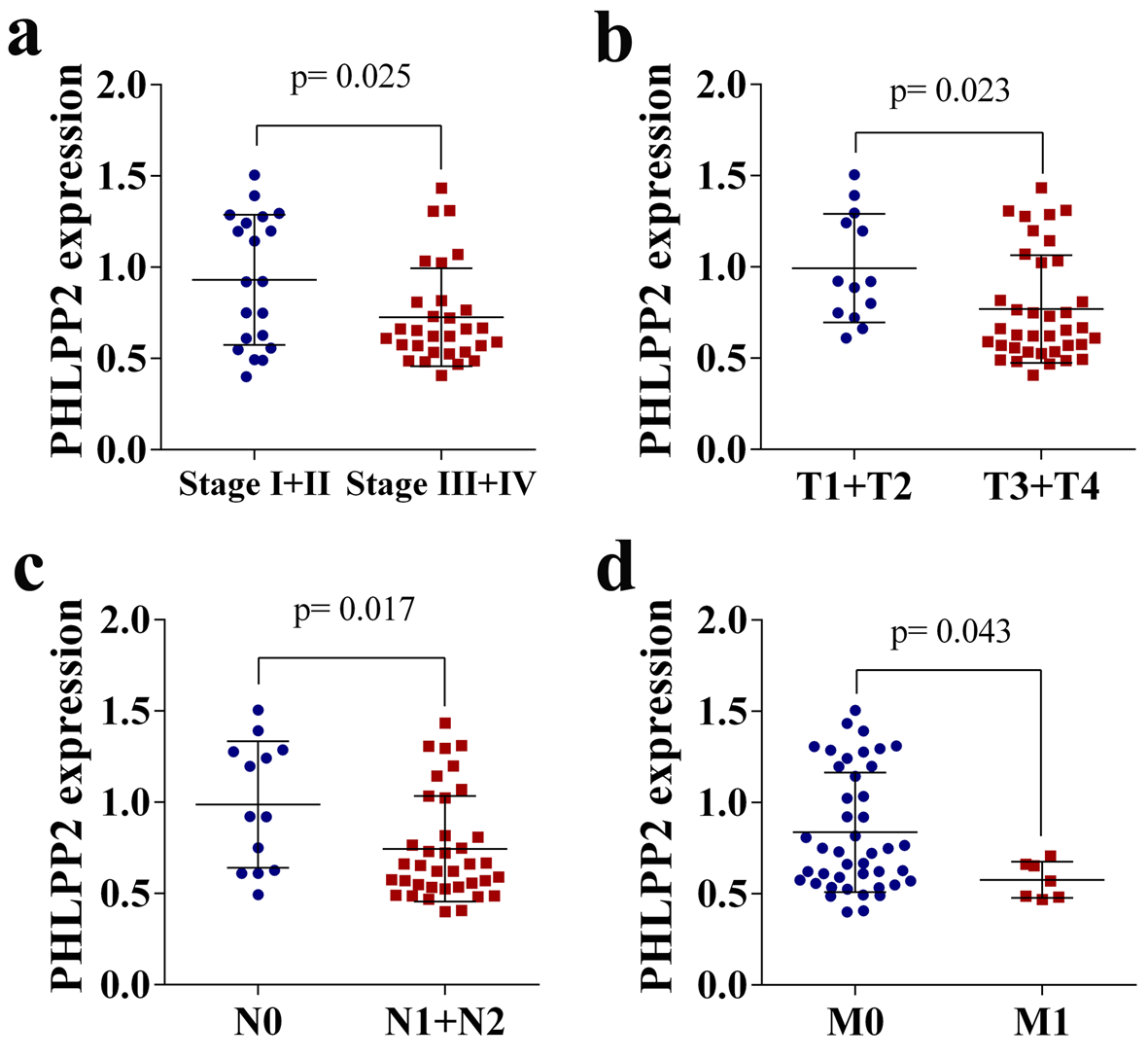
**

**Figure S2. Associations between PHLPP2 expression and clinicopathological features.** **a.** PHLPP2 expression in gastric cancers at different clinical stages. *P =* 0.025. **b.** PHLPP2 expression in gastric cancers at different T stages. *P =* 0.023. **c.** PHLPP2 expression in gastric cancers at different N stages. *P =* 0.017. **d.** PHLPP2 expression in gastric cancers at different M stages. *P =* 0.043.

**Figure S3:**

**
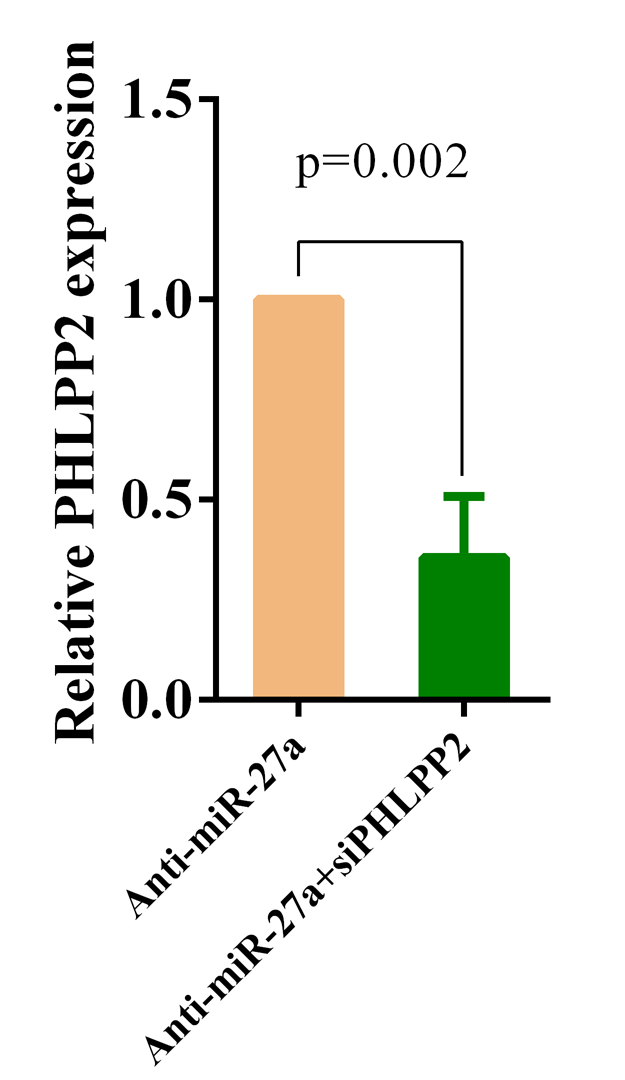
**

**Figure S3. Relative PHLPP2 mRNA expression level in SGC-7901 cells co-transfected with miR-27a antagomir and PHLPP2 siRNA.** PHLPP2 expression in SGC-7901 cells transfected with miR-27a antagomir and co-transfected with miR-27a antagomir/PHLPP2 siRNA was analyzed by qRT-PCR. *P =* 0.002.

**Figure S4:**

**
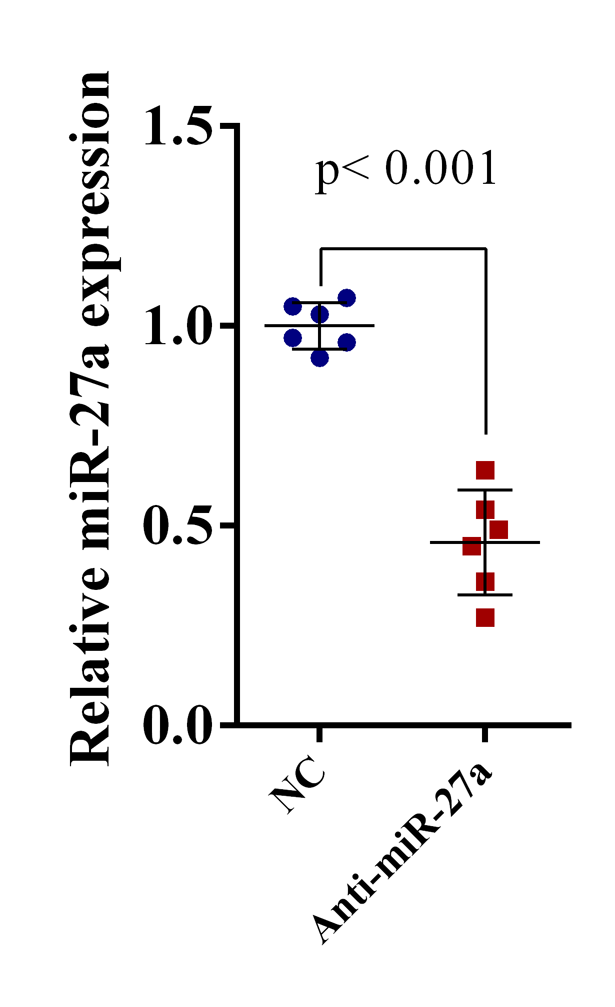
**

**Figure S4. Relative expression of miR-27a in subcutaneously trans-planted tumor tissues.** MiR-27a expression in xenografts treated with miR-27a antagomir and miR-27a antagomir NC. *P* < 0.001.
